# Chordin-Like 1 Improves Osteogenesis of Bone Marrow Mesenchymal Stem Cells Through Enhancing BMP4-SMAD Pathway

**DOI:** 10.3389/fendo.2019.00360

**Published:** 2019-06-12

**Authors:** Tao Liu, Bo Li, Xin-Feng Zheng, Sheng-Dan Jiang, Ze-Zhu Zhou, Wen-Ning Xu, Huo-Liang Zheng, Chuan-Dong Wang, Xiao-Ling Zhang, Lei-Sheng Jiang

**Affiliations:** ^1^Spine Center, Xinhua Hospital Affiliated to Shanghai Jiao Tong University School of Medicine, Shanghai, China; ^2^Department of Orthopedic Surgery, Xinhua Hospital Affiliated to Shanghai Jiao Tong University School of Medicine, Shanghai, China

**Keywords:** CHRDL1, hBMSCs, BMP-4, SMAD, RUNX2, osteogenic differentiation

## Abstract

Chordin-like 1 (CHRDL1) is a secreted glycoprotein with repeated cysteine-rich domains, which can bind to BMPs family ligands. Although it has been reported to play important roles in several systems, the exact roles of CHRDL1 on human bone mesenchymal stem cells (hBMSCs) osteogenesis remain to be explored. The present study aimed to investigate the roles of CHRDL1 on the osteogenic differentiation of hBMSCs and the underlying molecular mechanisms. We found that CHRDL1 was upregulated during hBMSCs osteogenesis, and rhBMP-4 administration could enhance CHRDL1 mRNA expression in a dose and time dependent manner. Knockdown of CHRDL1 did not affect hBMSCs proliferation, but inhibited the BMP-4-dependent osteogenic differentiation, showing decreased mRNA expression levels of osteogenic markers and reduced mineralization. On the contrary, overexpression of CHRDL1 enhanced BMP-4 induced osteogenic differentiation of hBMSCs. Moreover, *in vivo* experiments by transplanting CHRDL1 gene modified hBMSCs into nude mice defective femur models displayed higher new bone formation in CHRDL1 overexpression groups, but lower new bone formation in CHRDL1 knockdown groups, compared with control groups. In consistent with the bone formation rate, there were increased CHRDL1 protein expression in new bone formation regions of defective femur in CHRDL1 overexpression groups, while reduced CHRDL1 protein expression in CHRDL1 knockdown groups compared with control groups. These indicate that CHRDL1 can promote osteoblast differentiation *in vivo*. Furthermore, the mechanisms study showed that CHRDL1 improved BMP-4 induced phosphorylation of SMAD1/5/9 during osteogenic differentiation of hBMSCs. Besides, promotion of osteogenic differentiation and the activation of SMAD phosphorylation by CHRDL1 can be blocked by BMP receptor type I inhibitor LDN-193189. In conclusion, our results suggested that CHRDL1 can promote hBMSCs osteogenic differentiation through enhancing the activation of BMP-4-SMAD1/5/9 pathway.

## Introduction

Bone size and shape are precisely modeled and remodeled throughout life to ensure the structure and integrity of the skeleton (1). Bone remodeling is maintained by the regulation of two essential cell types, namely, the bone resorption osteoclasts and matrix-forming osteoblasts (2). Osteoporosis develops when the rate of osteoclastic bone resorption exceeds that of osteoblastic bone formation, which leads to loss of BMD and deterioration of bone structure and strength (3). Although osteoclast suppression machineries have been the focus of many bone studies, osteogenesis of BMSCs and its underlying mechanisms are also essential issues of bone remodeling (4).

Bone formation is mediated by osteoblasts recruited from bone mesenchymal cells (5), which can also differentiate into cells of other lineages, including myoblasts, chondrocytes, and adipocytes. The fate determination of bone marrow mesenchymal cells and their differentiation toward cells of the osteoblastic lineage is tightly controlled by several early regulators including: Wnt/β-catenin signaling, bone morphogenetic proteins (BMPs), hedgehog proteins, endocrine hormones, epigenetic regulators, and various growth factors. Among them, BMPs are known to exhibit high osteogenic activity (6).

BMPs usually function through BMPs-SMAD signaling pathway by binding to and signaling through types II and I BMP receptors, which are transmembrane serine/threonine kinases (7). Upon ligand binding, the types II(BMPR-II and ActR-IIA and ActR-IIB) and I(BMPR- I A or ALK-3,BMPRIB or ALK-6, and ActR- IA or ALK-2) receptors form a heterotetrameric complex (8), resulting in the phosphorylation of the type I receptor by the type II kinase domain. The phosphorylation of type I receptor facilitates the type I receptor to bind and phosphorylate SMAD1/5/9 proteins. Once phosphorylated, SMAD1/5/9 proteins form heterodimeric complexes with SMAD4 and translocate into the nucleus where they interact with other transcription factors, such as RUNX2, and stimulate the differentiation of BMSCs into osteoblasts (9). BMSCs committed to osteogenesis continue to develop the genetic profile and morphology of the osteoblast, expressing genes such as alkaline phosphatase, osteoprotegerin, type I collagen, and later osteocalcin (10). Osteogenic capability of BMPs, such as BMP-2 and BMP-7 have already been vastly studied and their recombinant proteins are currently being investigated in human clinical trials of craniofacial deformities, fracture healing, and spine fusion. However, several reports described the heterotopic ossification associated with their use which restricted their application (11, 12). It's imperative to further explore the osteogenic function and underlying regulation mechanism of other BMPs.

BMPs are functionally regulated by a class of intra and extracellular BMP-binding proteins, termed BMP antagonists, such as noggin, chordin, short gastrulation (Sog), twisted gastrulation (Tsg), and gremlin. BMP antagonists usually bind BMP family ligands and prevent their contact with receptors, thus inhibiting signaling. Chordin-like 1 (CHRDL1) is a secreted glycoprotein, which is structurally related to certain BMP antagonists and plays important roles in several systems, including angiogenesis (13), neural stem cell fate determination and neurogenesis (14), kidney protection from acute and chronic injuries (15), and suppression of tumor growth and metastasis(16, 17). Most of these functions are fulfilled by acting as BMP-4 antagonist. However, the role of CHRDL1 in human osteoblast differentiation induced by BMPs remains ambiguous. The intent of this study is to investigate the direct effect of CHRDL1 on human bone remodeling and the mechanisms involved.

## Materials and Methods

### Isolation and Expansion of Human BMSC (hBMSCs)

This study was approved by the Institutional Ethics Committee of Xin Hua Hospital Affiliated to Shanghai Jiao Tong University School of Medicine. Two male and two female patients averaged 45.7 years (range, 39–52 years) were recruited in this study. They all accepted traumatic femoral or tibia shaft fracture treatment by intramedullary nailing. Bone marrow samples from these patients were obtained with written consent, and patients presented with osteoporosis, other orthopedic or systemic diseases were excluded from the study.

Bone marrow blood aspirated during reaming from the femur or tibia of each donor was filtered through a 100 μm nylon mesh cell strainer. The filtrate was incubated in a 10 cm dish containing basal medium (BM) [low glucose Dulbecco's modified Eagle's medium (#SH30021.01; HyClone, USA) supplemented with 10% fetal bovine serum (#16000-044; Gibco, AUS), 100 U/mL penicillin G, and 100 mg/L streptomycin (#SV30010; HyClone, USA)] at 37°C in a humidified atmosphere containing 5% CO_2_. Non-adherent cells were discarded 72 h after cell-culture. Adherent cells were washed twice with phosphate buffered saline (PBS). Adherent hBMSCs cultured in complete medium were replaced every 2 days. When the cells achieved 90% confluence, the cultures were detached with 0.25% trypsin (#25200072; Gibco, AUS) and stored or reseeded for the following culture. Cells were used for subsequent experiments at passages 3 to 7.

### Identification of hBMSC Lineage

Approximately 4 ×10^5^ hBMSCs at passage 3 were incubated with 1 ug fluorescein isothiocyanate-conjugated mouse anti-human monoclonal antibodies at room temperature for 45 min. After washing with fluorescence-activated cell sorting (FACS) buffer (PBS with 10% bovine serum albumin and 1% sodium azide) at 376 g for 5 min, the stained cells were suspended in 250 μl of ice-cold FACS buffer and then analyzed with FACS (BD Biosciences, USA). For each sample, 1 ×10^4^ events were counted. The percentage of positive signals was analyzed using technical flow cytometry. The antibodies, including CD-29 (#555443; BD Pharmingen), CD-34 (#555822; BD Pharmingen), CD-44 (#555478; BD Pharmingen), CD-45 (#566156; BD Pharmingen), CD-90 (#551401; BD Pharmingen), and CD-105 (#323208; Biolegend), were used in this study.

### Multi-Lineage Differential Potential

For osteogenic differentiation assays, the culture-expanded cells were seeded at 1 ×10^5^ cells/well in a six-well culture plate and cultured in a complete culture medium until confluence. The cells were then cultured in BM or osteogenic medium(BM supplemented with 1 nM dexamethasone (#D4902; Sigma–Aldrich), 50 mM ascorbic acid(#A4403; Sigma–Aldrich), and 20 mM β-glycerolphosphate (#G9891; Sigma–Aldrich). The culture medium was changed every 3 days. The cells were cultured for 21 days for the assessment of Alizarin red S (#A5533; Sigma–Aldrich)staining.

For chondrogenic differentiation assays, culture-expanded cells were resuspended in BM at a density of 1 ×10^7^ cells/ml. A total of 20 ul cell suspension was carefully added to a 12-well plate. The cells were allowed to adhere at 37°C in 5% CO_2_ for 2 h, followed by the addition of 1 mL of BM or chondrogenic medium [BM supplemented with 10 ng/mL recombinant human transforming growth factor (TGF) beta 1 (#7666-MB-005; R&D Systems) and 50 ng/mL recombinant human insulin-like growth factor 1 (#6630-GR-025; R&D Systems)]. The culture medium was changed every 3 days. Micromasses were fixed for paraffin sectioning and Alcian blue staining after 28 days.

For adipogenic differentiation assays, culture-expanded cells were seeded at 1 ×10^5^ cells/well in a six-well plate and then cultured with BM. Upon reaching confluence, the medium was replaced with BM or adipogenic medium, wherein the BM was supplemented with 500 nM dexamethasone, 0.5 mM isobutylmethylxanthine (#I7018; Sigma–Aldrich), 50 mM indomethacin (#I7378; Sigma–Aldrich), and 10 mg/mL insulin (#I3536; Sigma–Aldrich). The medium was changed every 3 days. The cells were cultured for 14 days for oil red O (#O0625; Sigma–Aldrich) staining.

### siRNA Transfection

siRNA transfection was conducted using Lipofectamine 3000 (#L3000015;Thermo Fisher) according to the manufacturer's instruction. The sequences of siRNA targeting CHRDL1 (GenePharma Co., Ltd., Shanghai, China) were as follows: siRNA1: Sense 5′-GCAGCUGUUCGGAGGGAAATT dTdT-3′ and antisense 5′-UUUCCCUCCGAACAGCUGCTT dTdT-3′; siRNA2: Sense 5′-GCAAGCAUCAGGAACCAUUTT dTdT-3′ and antisense 5′-AAUGGUUCCUGAUGCUUGCTT dTdT-3′; siRNA3: Sense 5′-GCCUGUGUAUGAGUCUGUATT dTdT-3′ and antisense 5′-UACAGACUCAUACACAGGCTT dTdT-3′.

The sequences of negative control siRNA (NC-siRNA) that does not target any human gene product: 5′-UUCUCCGAACGUGUCACGUTT dTdT-3′ and anti-sense 5′-ACGUGACACGUUCGGAGAATT dTdT-3′.

### Lentiviral Transduction Overexpression Study

CHRDL1 gene was ligated into pLVX-IRES-puro to construct the CHRDL1 overexpression plasmid. The pLVX-IRES-puro and pLVX-IRES-puro-CHRDL1 were transfected into the HEK293T viral packaging cell line together with pSPAX2 and pMD 2.G. Exactly 48 h after transfection, the harvested cells were used for real-time PCR or Western blot analysis.

### ALP Staining

On the 7th day of osteogenic induction, ALP staining was performed. Cells were washed thrice with PBS and fixed with 4% paraformaldehyde for 15 min at room temperature. The samples were then stained with 0.1% naphthol AS-Biphosphate (Sigma-Aldrich, St. Louis, MO, USA) and 2% fast violet B (Sigma-Aldrich). After 30 min incubation at 37°C, the cell layer was washed thrice with deionized water to remove the dissociative dye and was observed under a digital camera.

### ALP Activity Assay

Cell layers were rinsed with PBS in triplicate and then lysated with lysis buffer containing 0.5% Triton, 50 mM of Tris–HCl, and 5 mM of MgCl_2_ (Sigma). ALP activity was assayed at 37°C in a buffer containing 0.1 M 2-amino-2-methyl-1-propanol (Wako), 2 mM MgCl_2_, and p-nitrophenyl phosphate (pNPP) for 30 min. The reaction was terminated by adding of 200 ml of 2 M NaOH per 200 μl of reaction mixture. Absorbance values were measured at 405 nm using pNPP (Sigma-Aldrich) as the substrate.

### Alizarin Red Staining and Calcium Assay

On the 14th day of osteogenic induction, cells were fixed in 75% ice-cold ethanol for 1 h and rinsed with distilled water. Cells were then stained with Alizarin Red S solution (Sigma) for 15 min until orange-red in color. After staining, the cells were washed thrice with deionized water, and observed under a digital camera. All experiments were repeated independently in triplicate.

### Cell Proliferation Assay

We performed CCK-8 assay according to the manufacturer's instructions. Serum-starved synchronized hBMSCs were seeded at 1 ×10^4^ cells/well in 96-well plates and cultured for 0, 24, 48 or 72 h and 10 ul Cell Counting Kit-8 solution (CCK-8; Dojindo Laboratories, Kumamoto, Japan) was added into the well. After 4 h incubation with CCK-8, cell proliferation was measured by reading optical density value at a wavelength of 450 nm.

### Real-Time PCR

Total RNA weighing about 1 ug was isolated using TRIzol reagent (Invitrogen) and reverse transcribed using the PrimeScript RT Master Mix cDNA Synthesis Kit (Takara, Japan) to obtain first strand cDNA. Real-time PCR was performed with a Roche LC 480 system using SYBR1 Premix (TaKaRa, Inc., Dalian, China) according to the instructions of the manufacturer. Glyceraldehyde 3-phosphate dehydrogenase (GAPDH) was used as an internal control. Data were analyzed using the comparison Ct (2^−ΔΔ*Ct*^) method and expressed as the fold change relative to GAPDH. Each sample was presented in mean with the standard error of triplicate.

Primer sequences were as follows: GAPDH: forward, 5′-AGGTCGGTGTGAACGGATTTG-3′; reverse, 5′-GGGGTCGTTGATGG CAACA-3′; CHRDL1: forward, 5′- CCTGGAACCTTATGGGTTGGT-3′; reverse, 5′-AACATTTGGACATCTGACTCGG-3′; ALP: forward, 5′-ACCACCACGAGAGTGAACCA-3′; reverse, 5′-CGTTGTCTGAGTACCAGTCCC; COL1A1: forward, 5′-GAGGGCCAAGACGAAGACATC-3′; reverse, 5′-CAGATCACGTCATCGCACAAC-3′; osteopontin (OPN): forward, 5′-CTGTGTTGGTGGAGGATGTCTGC-3′; reverse, 5′-GTCGGCGTTTGGCTGAGAAGG−3′; OCN: forward, 5′-GACAAGTCC CACACAGCAACT-3′; reverse, 5′-GGACATGAAGGCTTTGTCAGA-3′.

### Western Blot Analysis

Cells were washed with ice-cold Dulbecco's PBS and total protein lysates were extracted with cell lysis buffer RIPA (Biocolors, R0095) containing 1% PMSF (Meilunbio, MA0001). For western blot analysis, 20 ug of proteins was resolved on 10% SDS–PAGE gels (Bio-Rad, Richmond, CA) and transferred to polyvinylidene difluoride membranes (Merch, ISEQ00010).The membranes were blocked with TBS containing 5% (w/v) non-fat dry milk and 0.1% Tween-20 for 1 h, and then incubated at 4°C overnight with the appropriate antibodies, including CHRDL1 (Abcam, ab103369), BMPRII (CST, 6979), RUNX2 (Santa Cruz, 10758), p-SMAD1/5/9 (CST, 13820), SMAD1/5/9 (Santa Cruz, 6031), and GAPDH (CST, 5174). Blots were developed with horseradish peroxidase-labeled secondary antibody and visualized using the enhanced chemiluminescence detection system (Millipore, Billerica, MA) according to the manufactures' instructions.

### Surgical Procedure and Cell Transplantation

We used mice femoral shaft cortical bone defect model *in vivo* experiments, and all animal experiments were approved by the Laboratory Animal Institutions Committee. Animal care was provided in accordance with the Institutional Guidelines. Eight-week-old male BALB/C nude mice (Vital River Laboratory Animal Technology Co., Ltd. Beijing, China) were i.p. anesthetized with 1.5% pentobarbital sodium (40 mg/kg). A decimal bone defect 0.8 mm in diameter was performed on the femoral shafts. hBMSCs were suspended in the medium mixture and Matrigel (BD Bioscience), and 5–10^5^ cells/femoral shaft was transplanted into the defective lesions. hBMSCs were infected with si-CHRDL1 or pLVX- CHRDL1 before transplantation. Control mice underwent the same surgical operation except for transplantation of hBMSCs infected with pLVX-vector or NC-siRNA.

### Statistical Analysis

All statistical analysis was performed using SPSS (version 16.0; SPSS, Inc., Chicago, IL). All quantitative data were presented as the mean ± SD at least three separate experiments, each performed with triplicate samples and analyzed by Student's *t*-test or one-way ANOVA. All tests were two-sided with a *P-*value of 0.05 was used as the boundary of statistical significance.

## Results

### Identification and Characterization of hBMSCs

**Figure 1:**
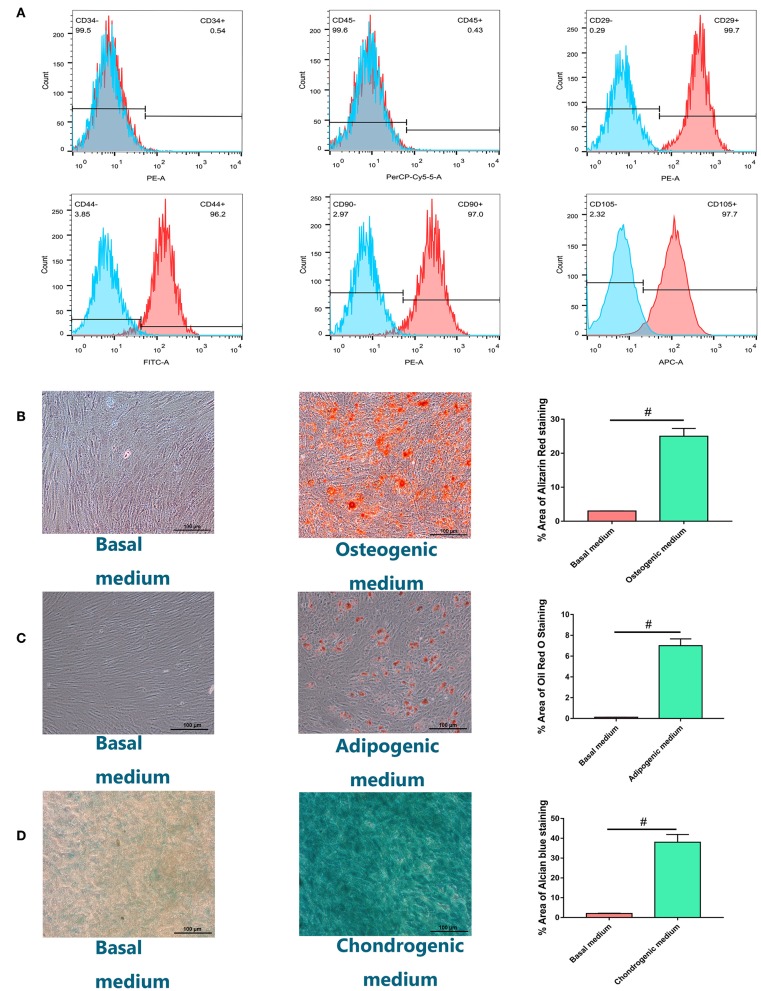
Identification and characterization of human bone marrow-derived mesenchymal stem cells (hBMSCs). **(A)** Flow cytometry analysis of the expression of positive (CD29, CD44, CD90, CD105) and negative (CD34, CD45) cell surface markers of hBMSCs. **(B)** Alizarin Red S staining for hBMSCs culturing for 21 days in osteogenic medium or basal medium. **(C)** Adipogenic differentiation potential of hBMSCs *in vitro*. Oil Red O staining for hBMSCs culturing for 14 days in adipogenic medium or basal medium. **(D)** Chondrogenic differentiation potential of hBMSCs *in vitro*. Alcian blue staining for hBMSCs culturing for 28 days in chondrogenic medium or basal medium with the method of micromass (magnification: ×100). Quantification of positively stained area recognized by image J was also shown in graph. All experiments were repeated independently in triplicate. Data were presented as mean ± SD (*n* = 3); ^#^*P* <0.01.

Isolated cells all expressed MSC markers CD29, CD44, CD90, and CD105 and did not express leukocyte and hematopoietic markers CD45 and CD34, respectively (Figure 1A). Most cells formed mineralized calcium deposits after 21 days of osteogenic differentiation, which were confirmed by Alizarin Red staining (Figure 1B). The adipogenic differentiation capacity of hBMSCs was confirmed by oil red O staining. Lipid droplets were detected 14 days after adipogenesis induction and were not observed in host medium (Figure 1C). After 28 days of micromass culture and induction, the cartilage differentiation of hBMSC was verified by positive staining with Alcian blue staining. Compared with chondrogenic induction in host medium, chondrogenic induction in micromass culture resulted in high Alcian blue staining (Figure 1D). Quantification of positively stained area recognized by image J was also shown in graph.

### The Expression of CHRDL1 Increased During Osteogenesis of hBMSCs

To understand the role of CHRDL1 during the process of osteogenesis, we determined the mRNA expression profile of CHRDL1 and early osteogenic marker ALP in hBMSCs cultured under osteogenic differentiation medium by using real-time PCR.

**Figure 2:**
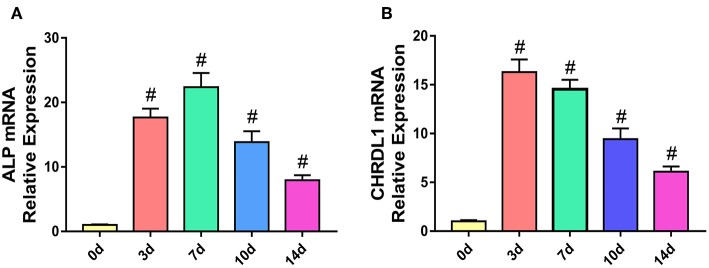
The expression models of CHRDL1 and ALP during the osteogenic differentiation of hBMSCs. hBMSCs were cultured with osteogenic medium, and total RNA was collected at 0, 3, 7, 10, and 14 days, and the mRNA expression levels of ALP **(A)** and CHRDL1 **(B)** were evaluated by real-time quantitative PCR. GAPDH was used as internal control. All experiments were repeated independently in triplicate. Data were presented as mean ± SD (*n* = 3); ^#^*P* <0.01 vs. 0 day.

During the process of osteogenic differentiation in hBMSCs, CHRDL1 mRNA expression, followed a similar distribution to that of ALP. CHRDL1 mRNA expression levels were detectable on day 0. During the first 7 days in culture, CHRDL1 expression levels peaked on day 3. During these days, the cells exhibited elevated ALP expression but the peak level appeared on day 7, which slightly lagged behind that of CHRDL1. CHRDL1 and ALP mRNA levels gradually declined on day 10 and then further declined to approximately half of the peak levels on day 14 (Figures 2A,B). These data showed that CHRDL1 gene was expressed in osteoblastic cells and its expression levels were regulated time dependently along with the osteogenic differentiation process.

### Suppression of CHRDL1 Decreased Osteogenesis of hBMSCs

**Figure 3:**
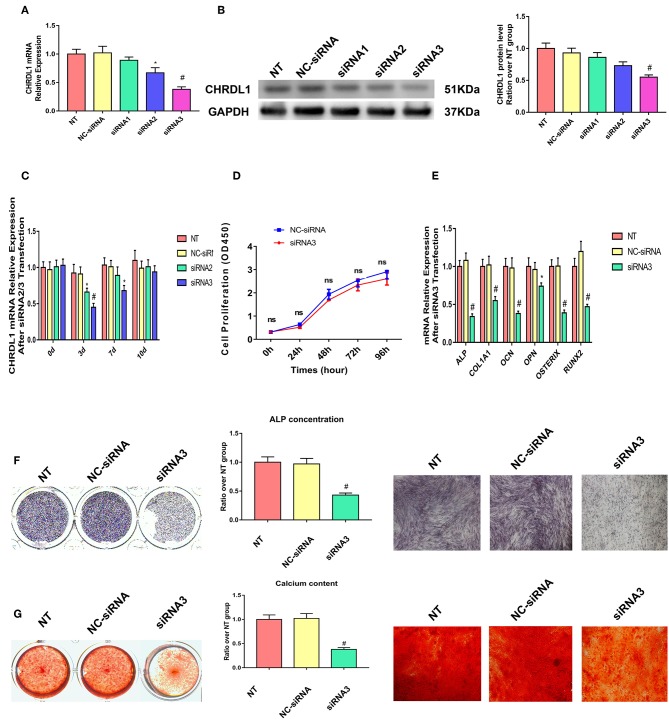
Knockdown of CHRDL1 suppressed hBMSCs osteogenesis. Three siRNAs were generated to suppress CHRDL1 expression. **(A,B)** CHRDL1 mRNA and protein expression levels were detected 72 h after siRNAs transfection. Densitometric analysis of immunoblot band intensities for CHRDL1 normalized by GAPDH were also detected. **(C)** CHRDL1 expression was detected at 0, 3, 7, and 10 days after siRNA2 or siRNA3 transfection. **(D)** *In vitro* growth of NC-siRNA and siRNA3 transfected hBMSCs were measured by CCK8 assay at 24, 48, 72, 96 h after transfection. **(E)** The mRNA expression levels of OCN, COL1A1, OPN, ALP, OSX, and RUNX2 were all detected using real-time quantitative PCR at 48 h after osteogenic induction and 72 h after siRNA3 transfection. **(F,G)** ALP staining, ALP quantitative analysis and Alizarin Red staining were performed to detect osteogenesis of hBMSCs after siRNA3 transfection. All experiments were repeated independently in triplicate. Data were presented as mean ± SD (*n* = 3) (*P <0.05 and ^#^*P* <0.01).

Three siRNAs were generated to suppress CHRDL1 expression to explore the possible function of CHRDL1 during osteogenic differentiation. Exactly 72 h after transfection, CHRDL1 siRNA2 and siRNA3 significantly reduced in corresponding CHRDL1 mRNA expression and protein levels (Figures 3A,B) in the culture supernatant compared with transfection of control siRNA. Transfection of CHRDL1 siRNA1 did not change CHRDL1 expression at mRNA and protein levels significantly. We conducted a time-course study with siRNA2 and siRNA3. Reduced CHRDL1 mRNA expression was observed in siRNA2- or siRNA3-transfected cells from day 0 to day 10 after transfection compared with non-transfected (NT) and control siRNA-transfected groups with the lower value that occurred 3 days after transfection by siRNA3 (Figure 3C). Given that siRNA3 achieved the best suppression effect on CHRDL1 expression, we conducted the following experiments with CHRDL1 siRNA3 only.

To make sure if CHRDL1 knockdown could affect hBMSCs proliferation, we conducted CCK8 assay according to the instructions. Fluorescence multi-well plate reader (Infinite M200 PRO, TECAN, Switzerland) with the optical density value set at a wavelength of 450 nm was used to detect siRNA3 and NC-siRNA transfected hBMSCs proliferation, no difference was detected at 24,48,72, 96 h after transfection between two groups (Figure 3D).

Exactly 24 h after transfection with CHRDL1 siRNA3, hBMSCs were induced by osteogenic medium without exogenous BMPs addition, and 72 h after transfection, the cells were harvested for quantitative PCR, mRNA expression levels of osteogenesis genes, such as ALP, OCN, OPN, COL1A1, OSTERIX, and RUNX2 were also significantly reduced compared with the control group (Figure 3E). ALP staining and ALP quantitative analysis showed that after 7 days of osteogenic induction, the transfection of CHRDL1 siRNA3 reduced the ALP activity of cells significantly (Figure 3F). Alizarin Red staining showed that the group transfected with CHRDL1 siRNA3 exhibited less calcium deposition than control groups. Similar results were obtained in photomicrographs (Figure 3G). A rescue experiment was performed by adding rhCHRDL1 protein (0.1 ug/ml) (Abcam, ab164881) to osteogenic medium 24 h after siRNA3 transfection. mRNA expression levels of osteogenesis related genes decreased by CHRDL1 suppression were largely rescued by rhCHRDL1 protein administration 72 h after transfection. ALP staining showed similar results after 7 days of osteogenic induction. (Supplementary Figures 1A,B).

### CHRDL1 Potentiate Osteogenesis Function of BMP-4

**Figure 4:**
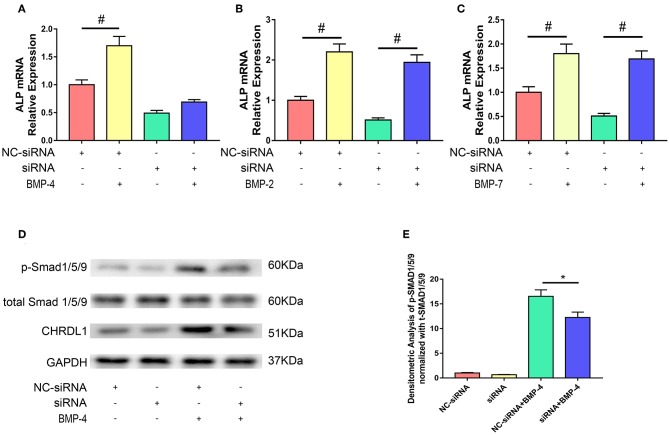
Knockdown of CHRDL1 inhibited BMP4-induced osteoblast differentiation of hBMSCs. hBMSCs were either transfected with si-CHRDL1 or NC-siRNA, 24 h later, both groups were induced by BMP-2, BMP-4, or BMP-7. 3 days after transfection, mRNAs were harvested for real-time quantitative PCR **(A-C)**. Western blotting was used to detect levels of CHRDL1, p-SMAD1/5/9, and total SMAD1/5/9 in hBMSCs transfected with NC-siRNA and si-CHRDL1 either with or without treatment with rhBMP-4 **(D)**. Relative levels of p-SMAD/t-SMAD were plotted graphically in panel **(E)**. The experiment was repeated three times. Data were reported as mean ± SE (*n* = 3) (*P <0.05 and ^#^*P* <0.01).

We next used the siRNA system to assess the possible role of CHRDL1 in the BMP-SMAD signaling pathway. hBMSCs were either transfected with si-CHRDL1 or NC-siRNA, 24 h later, both groups were induced by 0.1 ug/ml recombinant human BMP-2, BMP-4, and BMP-7 addition to test the dependence of BMPs function on CHRDL1. Three days after transfection, cells were harvested for quantitative PCR, rhBMP-4 administration can slightly rescue the decreased ALP mRNA level caused by CHRDL1 suppression (Figure 4A), whereas rhBMP-2 and rhBMP-7 substantially increased ALP mRNA levels (Figures 4B,C). Thus, we speculated that the induction of osteogenesis by BMP-4 depends on the presence of CHRDL1. Western blot was conducted to detect the level of SMAD-1/5/9 phosphorylation in response to rhBMP-4. Compared with cells transfected with NC-siRNA, hBMSCs that suppressed CHRDL1 by siRNA3, showed decreased p-SMAD-1/5/9 (Figure 4D) in a statistically significant fashion (Figure 4E). These data indicated that the function of BMP-4 was enhanced by and depended on the presence of CHRDL1 through BMP-SMAD signaling pathway during hBMSCs osteogenesis.

### Induced CHRDL1 mRNA Expression by BMP-4 Treatment

**Figure 5:**
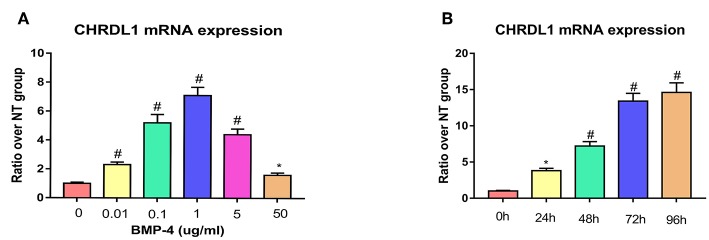
BMP-4 induced CHRDL1 expression in a time- and dose-dependent manner. **(A)** CHRDL1 mRNA expression of hBMSCs was tested using real-time quantitative PCR after treatment with indicated doses of rhBMP-4 for 72 h. *P <0.05 and ^#^*P* <0.01 vs. the group without BMP-4 treatment. **(B)** CHRDL1 mRNA expression of hBMSCs was tested with real-time quantitative PCR after treatment with 0.5 ug/ml rhBMP-4 for indicated time. All experiments were repeated independently in triplicate. Data were presented as mean ± SD (*n* = 3). *P <0.05 and ^#^*P* <0.01 vs. the CHRDL1 expression level at 0 h.

Given that the function of CHRDL1 on hBMSC osteogenesis was fulfilled via BMP-4, we next assessed the change of CHRDL1 mRNA expression during hBMSCs osteogenesis differentiation induced by 72 h rhBMP-4 treatment. The expression of CHRDL1 mRNA was induced by rhBMP-4 in a dose-dependent manner. In the range of 0–1 ug/ml rhBMP-4, increased concentrations of BMP-4 induced high expression of CHRDL1, and 1 ug/ml rhBMP-4 induced the peak expression. mRNA expression of CHRDL1 declined as the concentration of BMP-4 increased from 1 to 50 mg/ml (Figure 5A). CHRDL1 mRNA was also induced by BMP-4 in a time-dependent manner. The level of CHRDL1 mRNA induced by 0.5 ug/ml rhBMP-4 increased gradually with time and increased significantly at 48 and 72 h after the addition of rhBMP-4, and after 96 h the upward trend became gentle (Figure 5B).

Since CHRDL1 could be induced by BMP-4, we wonder if CHRDL1 expression could be blocked by BMP type I kinase inhibitor LDN-193189. We detected hBMSCs ALP and CHRDL1 mRNA expression levels after 72 h induction by rhBMP-4 with LDN-193189 administration. LDN-193189 (100 nM) significantly decreased ALP and CHRDL1 mRNA expression which was upregulated by rhBMP4 (Supplementary Figure 2).

### CHRDL1 Overexpression Enhances BMP-4 Induced Osteogenesis

**Figure 6:**
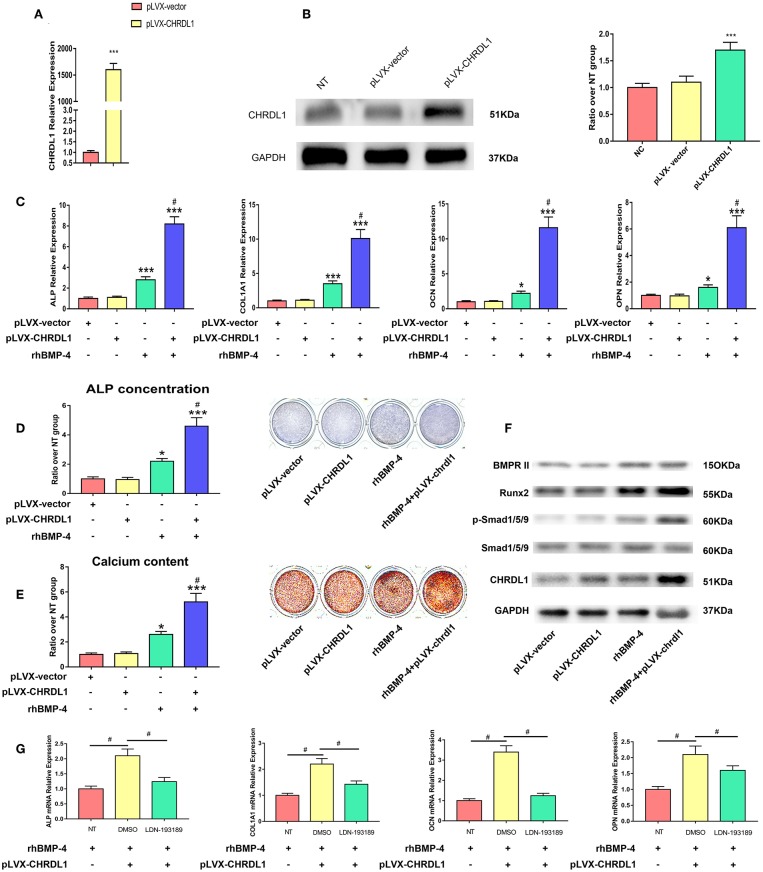
CHRDL1 overexpression enhanced BMP-4-induced osteoblast differentiation *in vitro*. **(A,B)** CHRDL1 mRNA expression and protein levels were detected at 72 h after transfection of pLVX-CHRDL1. ***P <0.01 compared with pLVX-vector. **(C)** The mRNA levels of COL1A1, ALP, OCN, and OPN were detected at 72 h after pLVX-CHRDL1 transfection and 48 h after osteogenic induction. *P <0.05; ***P <0.01 vs. pLVX-vector transfected sample; and ^#^*P* <0.01 vs. rhBMP-4 administrated sample. **(D,E)** ALP and Alizarin Red staining after transfection with pLVX-CHRDL1 and rhBMP-4 addition separately or in combination when cultured in osteogenic induction medium for 14 days. Data are presented as mean ±SD (*n* = 3). *P <0.05; ***P <0.01 vs. pLVX-vector transfected sample; and ^#^*P* <0.001 vs. rhBMP-4 administrated sample. **(F)** Western blot analysis of BMPR II, p-Smad1/5/9, total Smad1/5/9, Runx2, CHRDL1, and GAPDH at 48 h after transfection with pLVX-CHRDL1 and rhBMP-4 addition separately or in combination. GAPDH was used as loading control. **(G)** mRNA levels of ALP, COL1A1, OCN, and OPN were detected 48 h after treating with LDN-193189 or its vehicle (DMSO). Data were presented as mean ± SD (*n* = 3); (*P <0.05 and ^#^*P* <0.01). All experiments were repeated independently in triplicate.

We next examined whether CHRDL1 overexpression promoted osteogenic differentiation of hBMSCs. Lentivirus that expressed human CHRDL1 (pLVX- CHRDL1) under the control of the pLVX-vector were generated. pLVX-CHRDL1 was transduced into cultures of hBMSCs, and CHRDL1 mRNA and protein expression levels were analyzed by PCR and Western blot analysis 72 h after transfection. All these expressions increased substantially in a dose-dependent manner compared with cells transduced with control virus (pLVX-vector) (Figures 6A,B).

We then tested hBMSCs osteogenic differentiation affected by pLVX- CHRDL1 alone, BMP-4 administration, or by both. 24 h after transfection, hBMSCs were cultured in osteogenic differentiation medium, 48 h later, osteogenic marker expression was tested by real-time PCR. In cells transfected with pLVX-CHRDL1 alone, no significant effect on osteogenic marker expression was detected. However, 0.5 ug/ml rhBMP-4 application cells had increased osteogenesis and showed significantly higher ALP and COL1A1 mRNA levels and slightly higher late-osteogenic markers, such as OPN and OCN. Combined administration with rhBMP-4 and pLVX-CHRDL1 significantly enhanced ALP and COL1A1 mRNA levels over and above that of rhBMP-4 alone and also significantly increased mRNA expression of OPN and OCN (Figure 6C).

24 h after pLVX- CHRDL1 transfection, hBMSCs were induced for 7 or 21 days for ALP staining and Alizarin Red staining. Cells transfected with pLVX-CHRDL1 did not change the intensity of ALP and Alizarin Red staining compared with the control of the pLVX-vector, whereas rhBMP-4 administration significantly increased the staining, and they could increase further by combination of BMP-4 administration and pLVX-CHRDL1 transduction (Figures 6D,E). All these results confirmed our hypothesis that CHRDL1 acts to enhance BMP-4-mediated osteogenesis of hBMSCs.

To verify the relationship of CHRDL1 and BMP-4 during the osteogenic differentiation induced by them, we investigated the effect of CHRDL1 on downstream of the BMP-4-SMAD signaling pathway events during osteoblastic differentiation 48 h after pLVX-CHRDL1 transduction. BMP-4 singly application increased p-SMAD-1/5/9 level, and little such effect was observed in hBMSCs transfected with pLVX- CHRDL1. However, BMP-4 administration combined with pLVX- CHRDL1 transduction increased the phosphorylation of SMAD-1/5/9 in a statistically significant manner compared with BMP-4 alone (Figure 6F), indicating that CHRDL1 potentiates BMP-4 activity by increasing the activation level of SMAD-1/5/9.

*In vitro* gain of function experiment was also conducted with rhCHRDL1 addition instead of pLVX- CHRDL1 transfection. 0.1ug/ml rhCHRDL1 was added to the osteogenic medium 72 h for hBMSCs osteogenesis. Compared with control group, osteogenic related genes mRNA expression, ALP staining, as well as western blotting all showed results similar to those of pLVX- CHRDL1 transfection (Supplementary Figure 3). These results further confirmed osteogenic function of secreted glycoprotein CHRDL1.

To further confirm the functional connection between CHRDL1 and BMP-4, we examined the effect of LDN-193189 on pLVX- CHRDL1 transfection and rhBMP-4 administrated hBMSCs osteogenesis. 24 h after pLVX- CHRDL1 or pLVX-vector transfection, LDN193189 (100 nM) in its vehicle DMSO was applied during rhBMP-4 induced osteogenesis. 72 h after transfection, osteoblastic genes, such as COL1A1, ALP, OCN, and OPN mRNA expressions, were detected. Compared with pLVX-vector group, pLVX-CHRDL1 group showed higher mRNA levels, whereas descended mRNA levels were detected when treating with LDN-193189 (Figure 6G). All these data suggested that CHRDL1 enhanced BMP-4 function by increasing SMAD-1/5/9 phosphorylation level.

### *In vivo* Study

**Figure 7:**
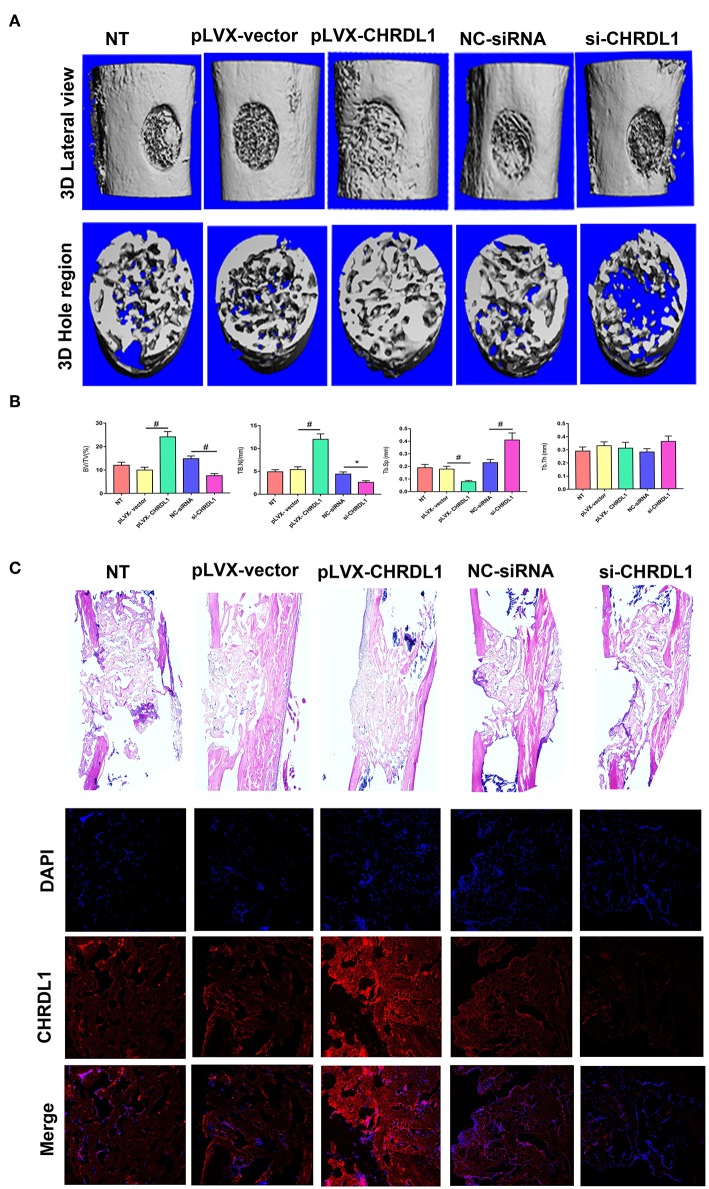
Knockdown and overexpression of CHRDL1 affected bone repair in a mouse model of femoral bone defect. **(A)** Lateral views of 3D reconstruction of defective femur (top panel) and mineralized bone formed in hole region (lower panel) by micro-CT. Representative images of each group. **(B)** 3D structural parameters of trabecular BV/TV, Tb.N, Tb.Sp, and Tb.Th of mineralized bone formed in hole region by micro-CT; **(C)** H & E staining also shows new bone accumulation in hole regions of si-CHRDL1 and pLVX-CHRDL1 treated mice. CHRDL1 expression detected by immunofluorescent assay around newly formed bone in each group were also shown. (Original magnification: 100 × ). All experiments were repeated independently in triplicate. (*P <0.05 and ^#^*P* <0.01).

To verify the role of gene CHRDL1 in osteogenesis *in vivo*, we performed two different experiments that used either a gain or loss of CHRDL1 function strategy. For gain of function experiment, hBMSCs transfected with pLVX- CHRDL1 or pLVX- vector were transplanted into the femoral defective lesions of immunocompromised mice. Two weeks after implantation, micro-CT showed that pLVX- CHRDL1 transduced cells generated more newly formed bone than cells that were transfected with pLVX-vector (Figure 7A). Percentage of bone volume (BV) to total tissue volume (TV) of callus was calculated to be significantly higher in pLVX- CHRDL1 group compared with both sham and pLVX- vector groups 3 weeks post-transplantation. Mineralized bone defects in pLVX-CHRDL1 group showed increased trabecular number (Tb.N), decreased trabecular separation (Tb.Sp), and unaltered trabecular thickness (Tb.Th) (Figure 7B). Newly formed bone detected by immunofluorescent assay showed that CHRDL1 expression in the pLVX- CHRDL1 group was higher than in the pLVX-vector group. consistently, H&E staining also showed increased new bone formation in the hole regions of defective femur of mice in PLVX- CHRDL1 group compared with the control groups (Figure 7C). These findings confirmed our previous *in vitro* experiments that showed CHRDL1 can enhance hBMSCs osteogenesis.

For loss of function experiment, hBMSCs transfected with si-CHRDL1 had dramatically reduced bone formation compared with cells transfected with NC-siRNA (Figure 7A) 2 weeks after implantation. si-CHRDL1 group showed significantly reduced percentage of BV to total TV, decreased Tb.N, increased Tb.Sp, and similar Tb.Th compared with two control groups 3 weeks post-transplantation (Figure 7B). CHRDL1 expression in the fibrous tissue around newly formed bone in the si-CHRDL1 group was lower and fewer new bone formation was also detected in H&E staining of si-CHRDL1 group (Figure 7C). Reduction in bone formation appeared to be due to a diminished ability of the si-CHRDL1 transfected hBMSCs to undergo differentiation, consistent with the reduction in the expression of osteogenesis markers, ALP, COL1A1, OCN, and OPN mRNA *in vitro* experiments.

We also conducted *in vivo* gain of function experiment by injecting rhCHDRL1 (0.1 ug/ml) mixed with Matrigel to the femoral defective lesions. Similar to the results of pLVX- CHRDL1 transfected hBMSCs transplantation, rhCHDRL1 local injection also significantly generated more new bone compared with the control group, assessed by micro-CT and HE staining 2 weeks after operation (Supplementary Figure 4).

To rule out the possibility of the secreted protein, CHRDL1, promote bone formation via affecting other cells such as: resident osteoblasts, osteoclasts and fibroblasts, immunohistochemical staining for Osteoprotegerin (OPG), tartrate-resistant acidic phosphatase (TRAP) and Gomori methenamine silver staining were conducted. No significant difference of stained area quantification in each experiment was detected between si-CHRDL1 and NC-siRNA group (Supplementary Figure 5), and these results may indicate these cell groups may not get involved in CHRDL1 potentiated osteogenesis.

## Discussion

Recent studies have advanced our understanding of the cellular events and signals that are involved in bone metabolism, and at the center of these advancements is the demonstration of the role of BMP signaling in skeletal biology (7). The BMP signaling pathway is highly conserved and vital to the development of various systems. Since the first BMP was discovered by Marshall Urist in the 1960s (18), more than 22 members of the BMP family have been identified to be critical molecules for osteoblastic activation and bone formation. These molecules are members of the TGF family, among them, BMP-4 is generally known for its critical roles in embryonic, hematopoietic, and mesenchymal developments, and has been identified as a regulator of cartilage and bone formation (19).

Although BMP-4 has been reported to work through other mechanisms (20), it usually functions through BMPs-SMAD signaling pathway. Many downstream molecules have been reported concerning bone formation in this signaling pathway, however, the complex regulation mechanism in several levels has not been fully understood. In addition to intracellular regulation, such as inhibitory SMADs, miRNAs, and methylation, extracellular regulation by “BMP antagonists” has been regarded as a pivotal morphogenetic mechanism of the BMPs-SMAD signaling pathway. BMP antagonists are a set of structurally distinct secreted proteins with repeated cysteine-rich (CR) domains, which bind to the BMP family ligands and prevent their contact with receptors, inhibiting BMP signaling. Secreted proteins with CR domains, such as chordin, Sog, Tsg, noggin, and gremlin-2, inhibit BMPs-SMAD signaling pathway via binding to BMP receptors (21). These observations indicate that BMP-antagonist expression is detrimental to bone formation. However, not all proteins containing CR domains are BMP inhibitors, some exert both stimulatory and antagonistic effects in different contexts. BMPER antagonizes or enhances BMP signaling, depending on the assay (22). Xiao also revealed that BMPER stimulates bone formation by coupling angiogenesis (23). Noggin plays varying roles during osteogenesis, inhibiting osteogenesis by preventing BMPs from binding to their receptors on the cell surface in some animal models, such as mice (24), and enhances osteogenesis by inducing BMP-2 and OCN in hBMSCs (25). Kielin/chordin-like protein is another BMP enhancer with CR domains, increasing the affinity of the ligand to the receptor and enhancing the stability of the ligand-receptor complex (26), further attenuating the pathology of renal fibrotic disease.

These studies extend our understanding of the role of “BMP antagonists,” revealing that proteins containing CR domain might also potentiate BMP-SMAD signaling and further benefit bone formation. CHRDL1 is a secreted glycoprotein containing three characteristic CR repeats structurally related to that of BMP antagonists and is reported to interact with several members of the BMP family, for example, BMP-4,−7, and−5 and TGF- β. CHRDL1 functions as a BMP antagonist in several systems, however, the exact role of CHRDL1 concerning osteogenesis is unclear. Hugo Fernandes et al. conducted an *in vitro* study, and showed that CHRDL1 upregulated hBMSC proliferation but unaffected osteogenic differentiation (27). However, hBMSC osteogenesis was induced only by BMP-2 in his study, whereas CHRDL1 was reported to interact with BMP-4 rather than BMP-2. Thus, their conclusion may be incomplete. Our results suggested that si-CHRDL1 did not affect hBMSCs proliferation but suppressed osteogenesis. And a stimulatory effect of CHRDL1 on osteogenesis of human BMSCs with the presence of BMP-4 was also detected. In addition, although hBMSCs with reduced CHRDL1 by knockdown showed increased phosphorylation of p-SMAD-1/5/9 in response to rhBMP-4 addition, phosphorylation level was significantly lower when compared with cells transfected with NC-siRNA, while CHRDL1 overexpression plus rhBMP-4 significantly upregulated SMAD1/5/9 phosphorylation, and RUNX2 protein level. Both loss and gain of function experiments consistently suggested that the induction of osteogenesis by BMP-4 was enhanced by and depended on the presence of CHRDL1. Furthermore, we noticed that BMP-4 induced CHRDL1 mRNA expression can be blocked by the addition of LDN193189, a specific BMP type I kinase inhibitor. The positive effect of combined administration of CHRDL1 and BMP-4 was also alleviated by LDN193189, these results further confirmed that osteogenesis of CHRDL1 was controlled by the BMP-4-SMAD pathway.

*In vitro*, single CHRDL1 overexpression or rhCHRDL1 addition did not significantly increase ALP activity and calcification as well as the expression of several osteoblastic genes, whereas combined addition of CHRDL1 and rhBMP-4 significantly promoted hBMSC osteogenesis. Whereas, *in vivo*, CHRDL1 could singly promote osteogenesis in femur bone defect models. The difference between *in vivo* and *in vitro* experiments may be explained by the presence of endogenous BMP-4 in bone defect models, which obviated the need for extra rhBMP-4 addition, indicating that CHRDL1 promotes osteogenesis depending on the presence of BMP-4.

In summary, the mechanism of CHRDL1 on bone formation may lie in a positive feedback loop and can be explained as follows. During hBMSCs osteogenesis, BMP-4 induced CHRDL1 expression, and BMP-4 activity was potentiated by CHRDL1. CHRDL1 further sensitized BMSCs to BMP-4, maintained the enhanced BMP-mediated signaling. As a CR domain containing “BMP antagonists,” CHRDL1 does not inactivate BMP-4 but rather acts as a novel inducer of hBMSCs osteogenesis through BMP4-SMAD signaling pathway although the possible roles of other BMPs in CHRDL1-regulated osteogenesis cannot be completely ruled out. Our study confirmed osteogenesis function of rhCHRDL1 *in vivo*, which lays a foundation for its clinical application, although its systemic or local concentrations need to be further studied to understand the diagnostic and therapeutic potential of CHRDL1.

In conclusion, our study indicated that osteogenesis induction by BMP-4 was enhanced by and depended on the presence of CHRDL1. The ability of CHRDL1 to enhance BMP-4 activity might be an important mechanism to elucidate the mechanisms of hBMSCs osteogenic differentiation and bone remodeling, and CHRDL1 might be a potential treatment target for metabolic and developmental bone diseases.

## Author Contributions

TL designed and conducted the study, contributed to the data analysis, and writing the manuscript. BL collected and analyzed data. S-DJ and X-FZ supervised the statistical analysis and contributed to writing the manuscript. Z-ZZ, W-NX, H-LZ, and C-DW wrote and revised the paper. L-SJ and X-LZ supervised this study and contributed to study design, data analysis, and writing the manuscript.

### Conflict of Interest Statement

The authors declare that the research was conducted in the absence of any commercial or financial relationships that could be construed as a potential conflict of interest.

## Supplementary Material

Supplementary Figure 1 rhCHRDL1 rescued hBMSCs osteogenesis suppressed by si-CHRDL1. **(A)** mRNA expression levels of osteogenesis related genes and were detected at 72 h after si-CHRDL1 transfection and 48 h after rhCHRDL1 protein (0.1 ug/ml) administration. **(B)** ALP staining was detected after 7 days of osteogenic induction. Data were presented as mean ± SD (*n* = 3); ^*^*P* <0.05; ^***^*P* <0.001 vs. NC-siRNA transfected sample; and ^#^*P* <0.001 vs. siRNA3 transfected sample. All experiments were repeated independently in triplicate.

Supplementary Figure 2 BMP type I kinase inhibitor LDN-193189 blocked ALP and CHRDL1 mRNA expression upregulated by rhBMP4. ALP **(A)** and CHRDL1 **(B)** mRNA expression levels were detected after 72 h rhBMP-4 induction in combination with LDN-193189 administration. Data were presented as mean ± SD (*n* = 3); (^#^*P* <0.01). All experiments were repeated independently in triplicate.

Supplementary Figure 3 rhCHRDL1 enhanced BMP-4-induced osteoblast differentiation *in vitro*. Osteogenesis related gene mRNA expression levels **(A)** and ALP staining **(B)** after addition of rhCHRDL1 and rhBMP-4 addition separately or in combination when cultured in osteogenic induction medium for 72 h. Data were presented as mean ± SD (*n* = 3); ^*^*P* <0.05; ^***^*P* <0.01 vs. NC group sample; and ^#^*P* <0.01 vs. rhBMP-4 administrated sample. **(C)** Western blot analysis of BMPR II, p-Smad1/5/9, total Smad1/5/9, Runx2 and GAPDH at 72 h after rhCHRDL1 and rhBMP-4 addition separately or in combination. GAPDH was used as loading control. All experiments were repeated independently in triplicate.

Supplementary Figure 4 rhCHRDL1 addition promoted bone repair in a mouse model of femoral bone defect. **(A)** Representative images of lateral views of 3D reconstruction of defective femur and mineralized bone formed in hole region by micro-CT. **(B)** H & E staining also shows new bone accumulation in hole regions of control group and rhCHRDL1 treated mice. (Original magnification: 100 × ). All experiments were repeated independently in triplicate.

Supplementary Figure 5 Knockdown of CHRDL1 did not affect osteoblasts, osteoclasts and fibroblasts in femoral bone defect model. Quantification of positively stained area of of OPG staining **(A)**, TRAP staining **(B)**, and Gomori methenamine silver staining **(C)** recognized by image J was also shown in graph. All experiments were repeated independently in triplicate.
