# Supplementary figures and images for: Chordin-Like 1 Improves Osteogenesis of Bone Marrow Mesenchymal Stem Cells Through Enhancing BMP4-SMAD Pathway
Source: Front Endocrinol (Lausanne). 2019 Jun 12;10:360. doi: 10.3389/fendo.2019.00360 (PMC6582276; doi:10.3389/fendo.2019.00360)

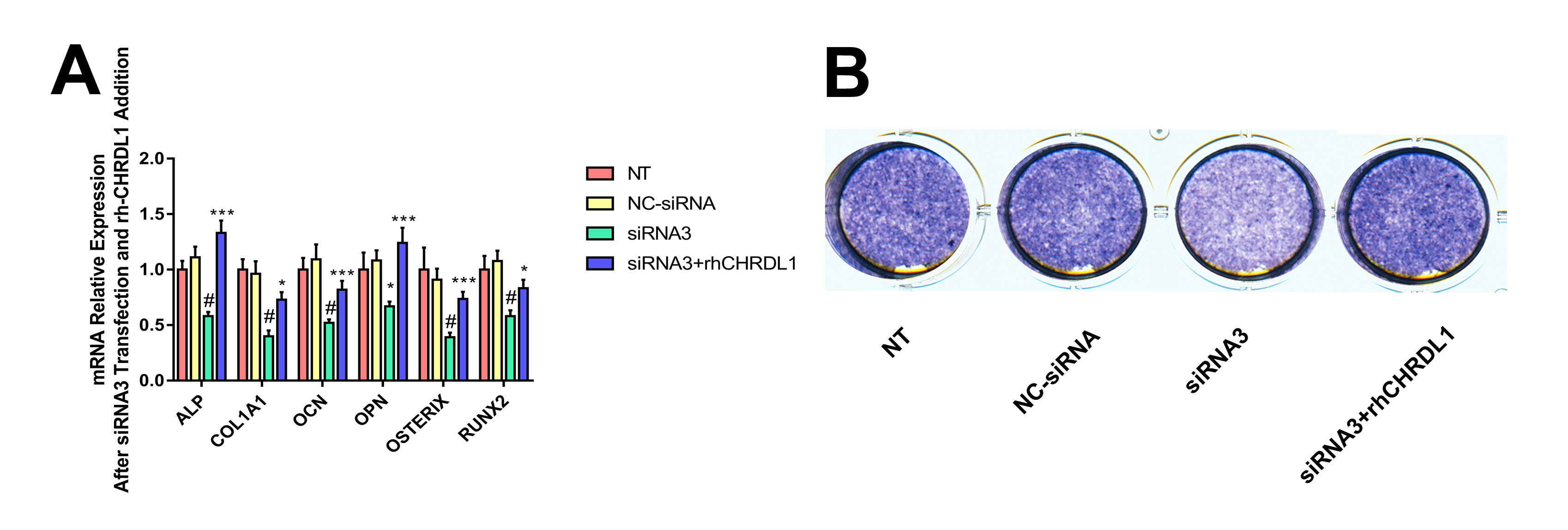

Supplement: Supplementary Figure 1 — rhCHRDL1 rescued hBMSCs osteogenesis suppressed by si-CHRDL1. (A) mRNA expression levels of osteogenesis related genes and were detected at 72 h after si-CHRDL1 transfection and 48 h after rhCHRDL1 protein (0.1 ug/ml) administration. (B) ALP staining was detected after 7 days of osteogenic induction. Data were presented as mean ± SD (n = 3); *P <0.05; ***P <0.001 vs. NC-siRNA transfected sample; and #P <0.001 vs. siRNA3 transfected sample. All experiments were repeated independently in triplicate. [file Image_1.JPEG]

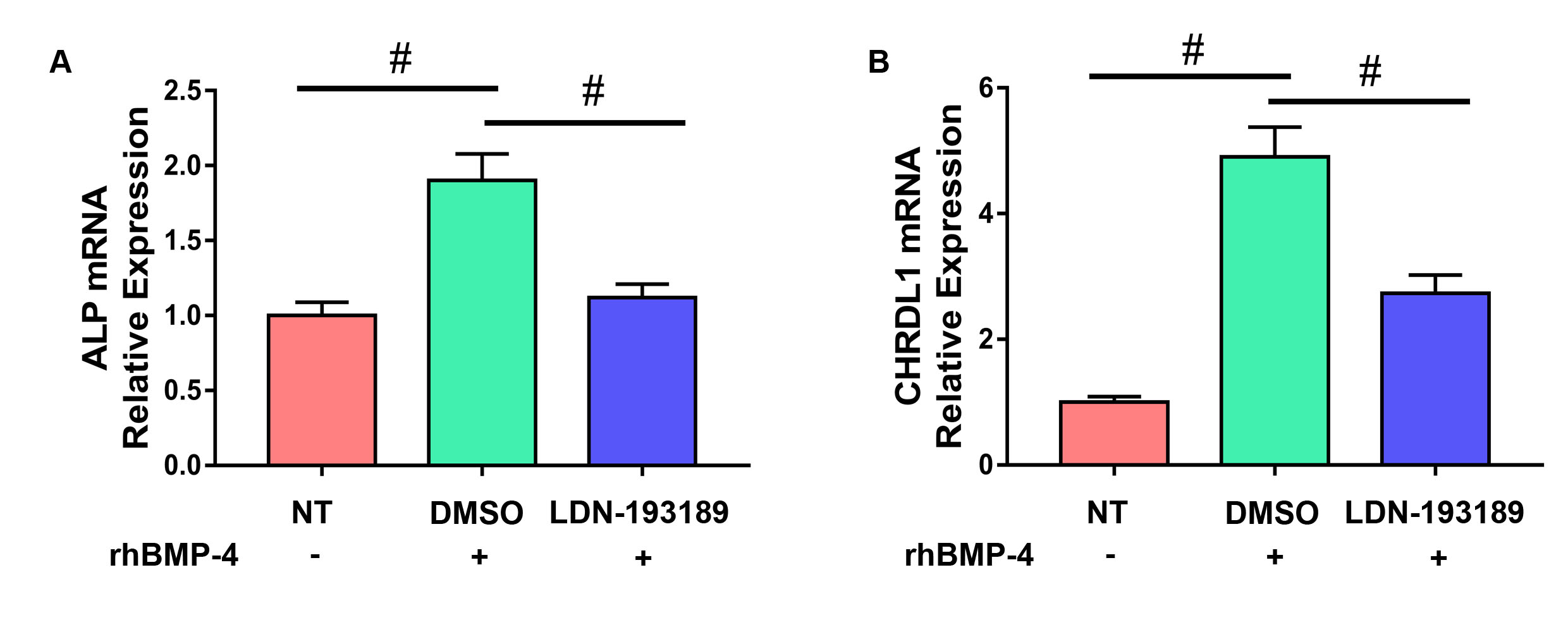

Supplement: Supplementary Figure 2 — BMP type I kinase inhibitor LDN-193189 blocked ALP and CHRDL1 mRNA expression upregulated by rhBMP4. ALP (A) and CHRDL1 (B) mRNA expression levels were detected after 72 h rhBMP-4 induction in combination with LDN-193189 administration. Data were presented as mean ± SD (n = 3); (#P <0.01). All experiments were repeated independently in triplicate. [file Image_2.JPEG]

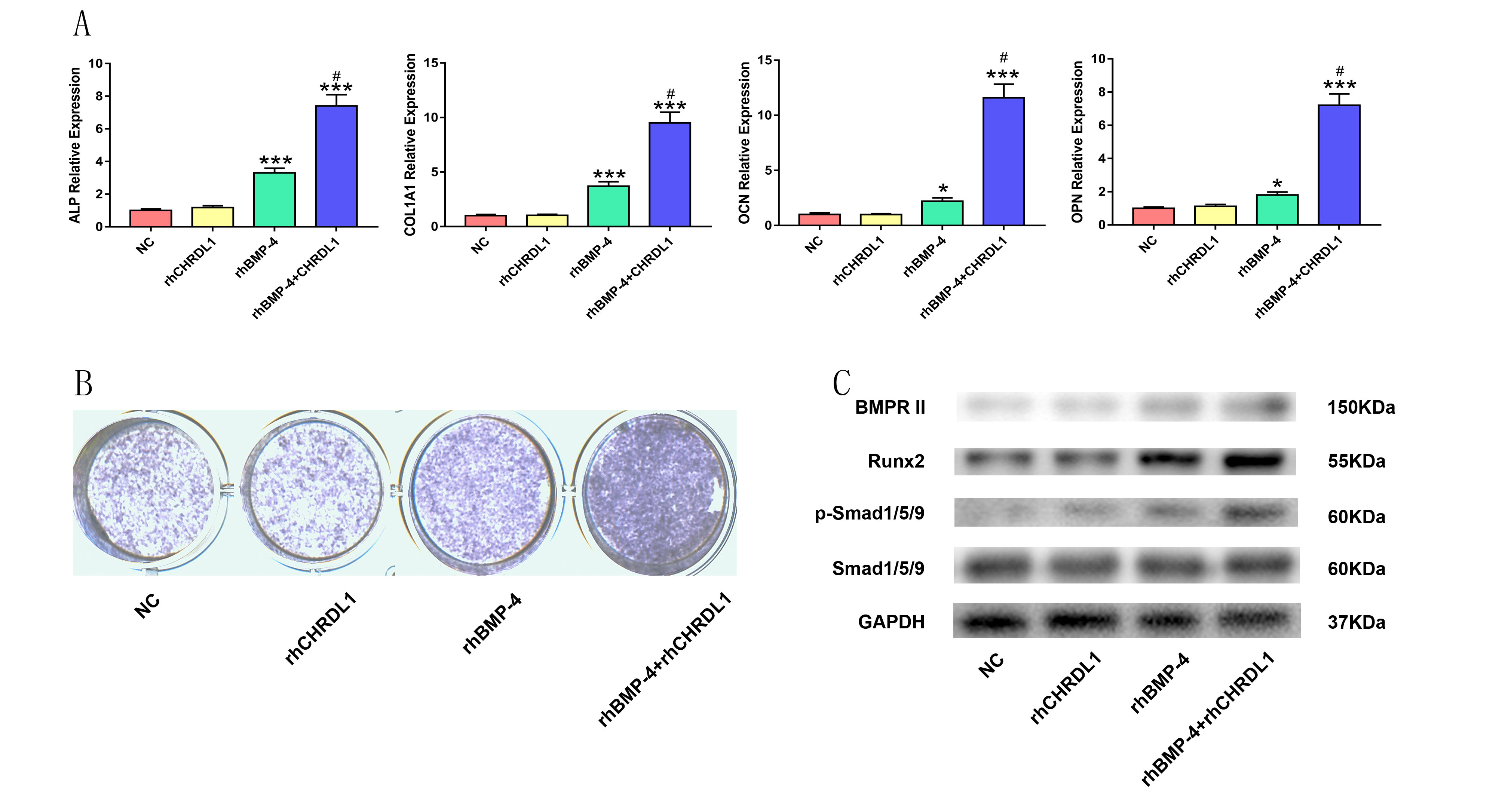

Supplement: Supplementary Figure 3 — rhCHRDL1 enhanced BMP-4-induced osteoblast differentiation in vitro. Osteogenesis related gene mRNA expression levels (A) and ALP staining (B) after addition of rhCHRDL1 and rhBMP-4 addition separately or in combination when cultured in osteogenic induction medium for 72 h. Data were presented as mean ± SD (n = 3); *P <0.05; ***P <0.01 vs. NC group sample; and #P <0.01 vs. rhBMP-4 administrated sample. (C) Western blot analysis of BMPR II, p-Smad1/5/9, total Smad1/5/9, Runx2 and GAPDH at 72 h after rhCHRDL1 and rhBMP-4 addition separately or in combination. GAPDH was used as loading control. All experiments were repeated independently in triplicate. [file Image_3.JPEG]

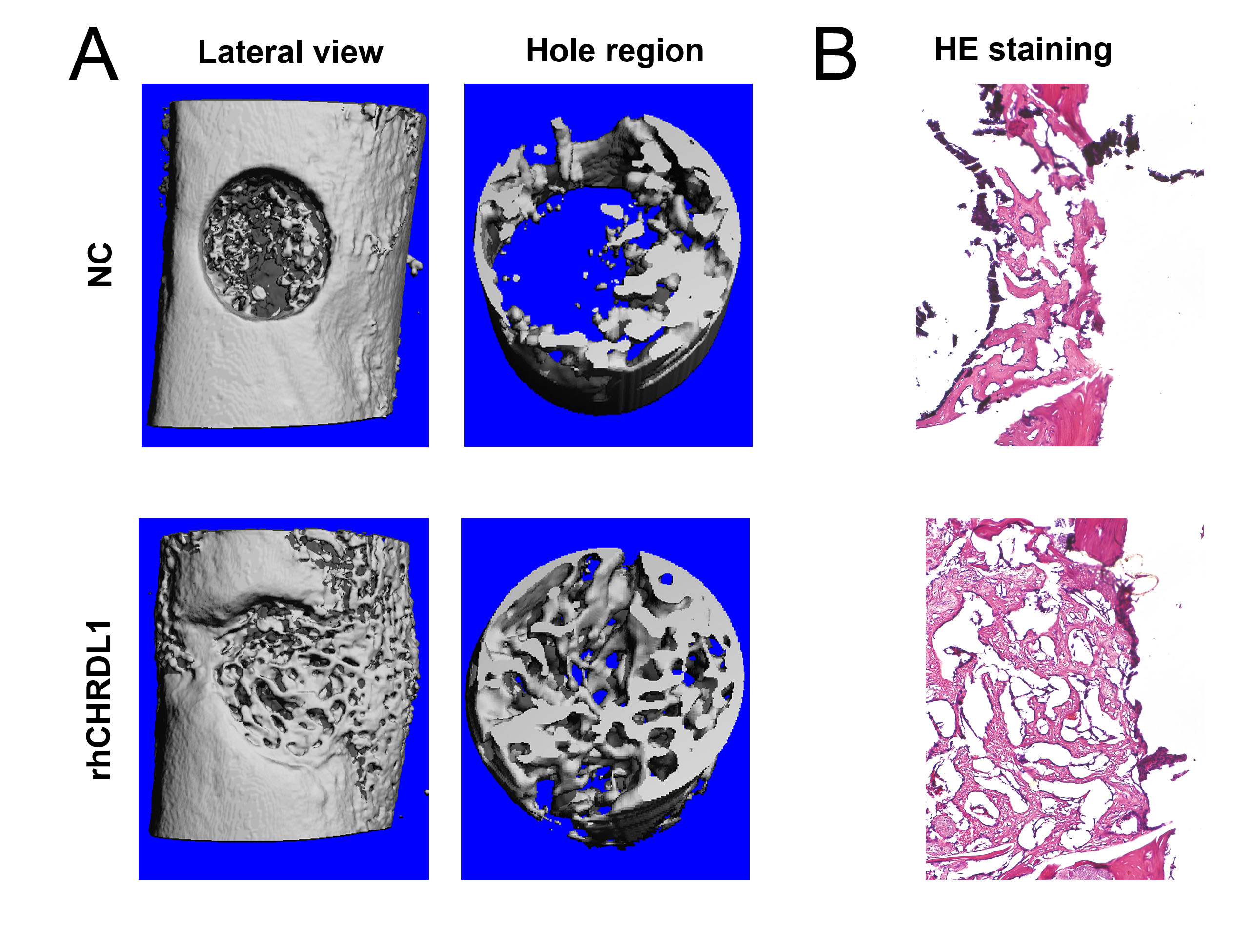

Supplement: Supplementary Figure 4 — rhCHRDL1 addition promoted bone repair in a mouse model of femoral bone defect. (A) Representative images of lateral views of 3D reconstruction of defective femur and mineralized bone formed in hole region by micro-CT. (B) H & E staining also shows new bone accumulation in hole regions of control group and rhCHRDL1 treated mice. (Original magnification: 100 × ). All experiments were repeated independently in triplicate. [file Image_4.JPEG]

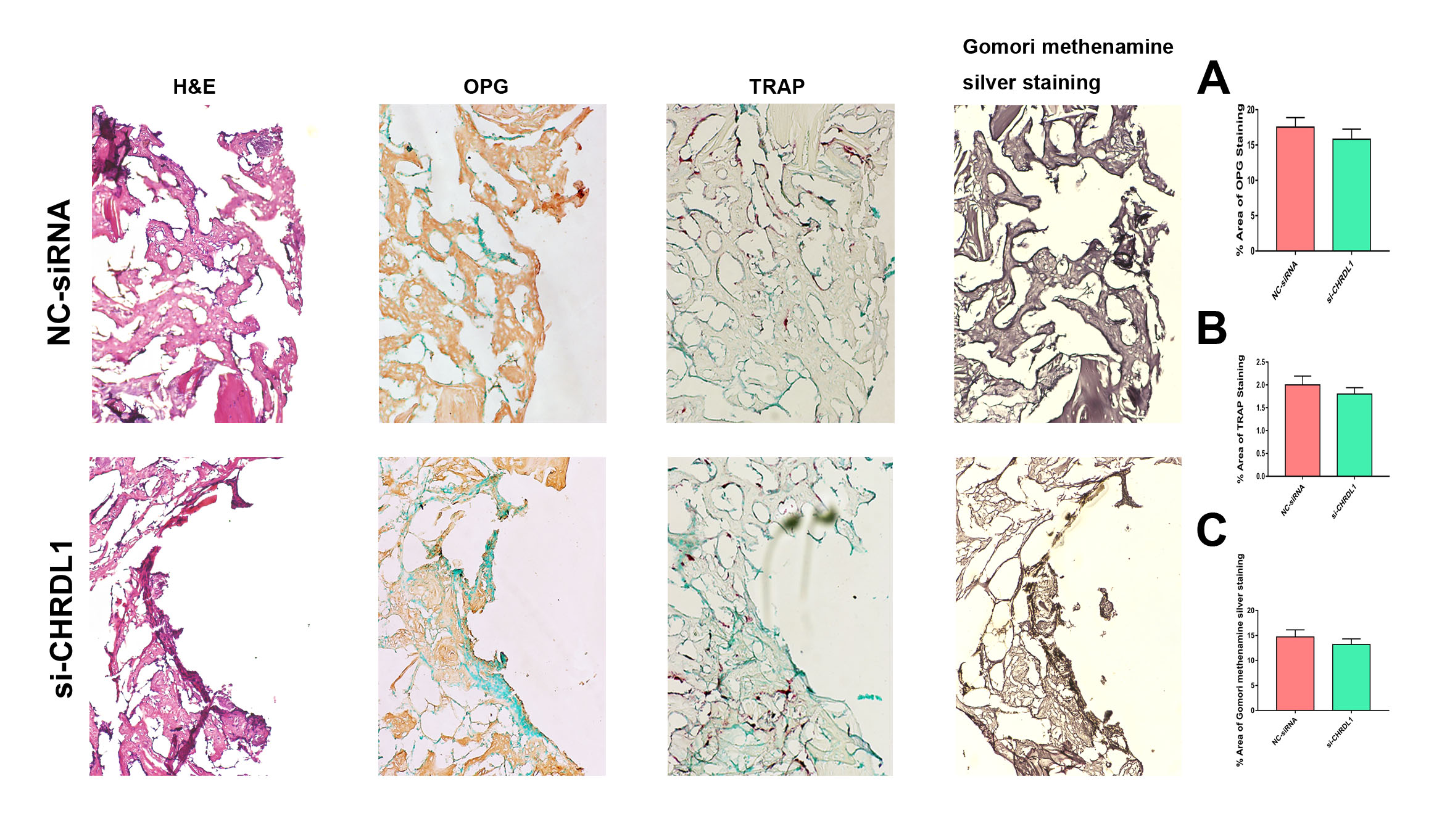

Supplement: Supplementary Figure 5 — Knockdown of CHRDL1 did not affect osteoblasts, osteoclasts and fibroblasts in femoral bone defect model. Quantification of positively stained area of of OPG staining (A), TRAP staining (B), and Gomori methenamine silver staining (C) recognized by image J was also shown in graph. All experiments were repeated independently in triplicate. [file Image_5.JPEG]
